# Distress as a bridge to suicidality in schizophrenia spectrum disorders: a network-based intervention simulation study

**DOI:** 10.21203/rs.3.rs-8855938/v1

**Published:** 2026-02-18

**Authors:** Jinyuan Liu, Fay Womer, Julia Sheffield, Kristan Armstrong, Trey McGonigle, Jennifer Blackford, Neil Woodward, Stephan Heckers, Brandee Feola

**Affiliations:** Vanderbilt University Medical Center; Vanderbilt University Medical Center; University of Nebraska Medical Center; Vanderbilt University Medical Center; Vanderbilt University; Vanderbilt University Medical Center

## Abstract

Schizophrenia spectrum disorders (SSD) are associated with a markedly elevated risk of suicide, yet the symptom-level mechanisms linking psychopathology to suicidality remain incompletely understood. Network-based models provide a data-driven framework for characterizing symptom interrelations, identifying candidate intervention targets, and generating hypotheses about potential downstream effects. However, the Positive and Negative Syndrome Scale (PANSS), the gold-standard assessment of schizophrenia symptoms, has primarily been used as a static rating tool rather than as a dynamic simulation framework for intervention prioritization. Here, we developed a network-based simulation framework using PANSS to identify symptom domains most strongly associated with suicidality and to evaluate how targeted modulation of these symptoms may relate to suicidal ideation. Symptom networks reproduced a robust five-domain PANSS structure (Positive, Negative, Cognitive Impairment, Impulsive-Hostile, and Distress). Across item- and cluster-level analyses, distress-related symptoms (anxiety, depression, guilt, and tension) showed the most consistent associations with suicidality and emerged as key bridge symptoms. Analyses stratified by lifetime suicide attempt history revealed distinct networks and simulation responses across risk strata. Simulation-based modulation of the Distress domain was associated with attenuated network connectivity and lower predicted suicidal ideation. While derived from simulations, these findings highlight affective distress as a clinically relevant symptom pathway linked to suicide risk in SSD and demonstrate how network-informed digital simulations may support the prioritization of symptom targets. Future studies, particularly randomized controlled trials, will be critical to determine whether targeting distress leads to measurable reductions in suicidality, with simulation-based analyses complementing observational evidence by informing hypothesis generation and trial design.

## Introduction

Schizophrenia spectrum disorders (SSD) affect approximately 1% of the population and are associated with profound personal, familial, and societal burdens (1–3). Among the most devastating consequences of schizophrenia spectrum disorders is the markedly elevated risk of suicide (4). Suicide accounts for nearly half of premature mortality in schizophrenia spectrum disorders, with lifetime rates of suicide estimated at 4–13% and suicide attempts at 18–55% (5, 6). The risk is particularly pronounced among individuals with a prior suicide attempt (7, 8). More specifically, people with SSD with a lifetime history of suicide attempt show a three- to five-fold increased risk for future suicidal behavior (57, 58). However, the mechanisms linking SSD symptomology to suicidality remain insufficiently understood, posing a critical barrier to the development of targeted suicide-prevention interventions (4, 9).

Despite decades of research, consistent predictors of suicide risk in schizophrenia spectrum disorders remain elusive. Some risk factors overlap with those observed in the general population, such as major life events and unemployment (5, 10), yet SSD confer additional disorder-specific risk factors for suicide (7, 8). Notably, the association between psychotic symptoms and suicidality is unclear as findings have been mixed, with no consistent association between positive or negative symptoms and suicide risk (11, 12). By contrast, alterations in negative affect have consistently emerged as more robust risk factors of suicidal behavior in individuals with SSD (7, 13–15). Importantly, these affective symptoms, including depression, anxiety, hopelessness, and related distress symptoms, appear to be particularly salient among individuals with a prior suicide attempt, suggesting that suicide risk may arise through distinct symptom pathways. However, the interplay between negative affect and the increased risk for suicide remains poorly understood, and it is unknown whether particular symptoms serve as mechanistic “gateways” through which suicidality may be attenuated (16).

Network analyses provide a powerful data-driven framework for conceptualizing psychopathology as a mutually reinforcing system. Network models quantify how disturbances in one symptom domain may influence others by representing symptoms as nodes connected through partial correlations (17–20). Centrality metrics can be used to identify specific symptoms that exert strong influences within the network (e.g., highly interconnected), thereby highlighting potential targets for intervention (21). When suicidality is incorporated as an additional node, network models can further identify “bridge” symptoms that directly link psychopathology to suicidal ideation, thereby offering a mechanistic perspective on suicide risk and informing targeted prevention strategies (22–24).

Network approaches are particularly well-suited for SSD, which exhibit heterogeneity across symptom domains (19, 25–28). The Positive and Negative Syndrome Scale (PANSS), the most widely used clinician-rated instrument in schizophrenia research and clinical trials, demonstrates strong psychometric properties (e.g., Cronbach’s Alpha > 0.80; inter-rater reliability > 0.80) and captures core symptom dimensions relevant to suicide risk (29–32). Using PANSS data, recent large-scale longitudinal network studies have identified potential bridge symptoms linking core psychopathology to suicidality, including depression and conceptual disorganization (24). While network analyses characterize the structure of symptom interrelations, simulation-based intervention modeling extends this framework by evaluating hypothetical perturbations to specific symptoms and propagating their effects through the network. Building on this literature, the present study advances a translational framework that leverages such simulations to prioritize symptom targets for empirical evaluations and inform the design of future clinical trials (33–36).

Leveraging PANSS-based symptom networks, the present study aims to identify intervention targets most strongly associated with suicidality in individuals with SSD. We first construct PANSS symptom networks and incorporate suicidality as a node to identify bridge symptoms linking psychopathology to suicidal ideation. We then evaluate candidate symptoms using network centrality metrics and network bridges to assess their potential as intervention targets. Recognizing that individuals with a lifetime history of suicide attempt represent a clinically distinct subgroup characterized by enduring vulnerability, we explicitly stratify analyses by suicide attempt history to examine whether symptom networks and their response to targeted interventions differ across risk strata. Finally, we conduct simulation-based interventions to assess whether modulating these candidate symptoms predicts reductions in suicidality. Based on prior evidence, we hypothesize that distress-related symptoms, including depression, anxiety, guilt, and tension, will emerge as key targets for suicide prevention in this population.

## Methods

### Participants

The study consisted of 362 individuals aged 18–65 years with SSD: schizophrenia (SZ; *N* = 154), schizoaffective disorder (SZA; *N* = 78), and schizophreniform disorder (SZF; *N* = 130). Diagnoses were confirmed using the Structured Clinical Interview for DSM-IV or DSM-5 (37). Participants were recruited from the Psychotic Disorders Program at Vanderbilt Psychiatric Hospital (Nashville, TN).

Exclusion criteria included estimated premorbid IQ < 70, major systemic medical illness, central nervous system disorders, significant head trauma, or substance use disorder within the preceding three months (38). All participants provided written informed consent and received compensation. The study was approved by the Vanderbilt Institutional Review Board and used data from a previously described cohort (27).

## Clinical Measures

### Psychosis Symptoms

All participants were assessed by the Positive and Negative Syndrome Scale (PANSS), a clinician-rated, 30-item interview assessing symptom severity over the preceding week (30). Items are scored from 1 (“absent”) to 7 (“extreme”). PANSS items span Positive, Negative, Cognitive, Hostility, and Mood/Distress domains (39–41), which provide clinically meaningful coverage of schizophrenia symptomatology.

### Suicidality

Suicidality was assessed using two complementary measures: lifetime history of *suicide attempt* and current *suicidal ideation*. Lifetime suicide attempt (SA) history was available for 317 participants and was assessed using an in-house, interview-rated instrument directly following the Structured Clinical Interview for DSM-IV or DSM-5 (SCID) (see **Supplement**). When available, hospital and outpatient medical records were reviewed to corroborate the self-report. In the present analyses, we used the binary indicator of lifetime suicide attempt history, which was used as a stratification factor reflecting a trait-like vulnerability to suicidal behavior (42).

A subset of 313 participants (138 SZ, 69 SZA, 106 SZF) completed the Montgomery-Asberg Depression Rating Scale (MADRS), an interviewer-administered measure of depressive symptom severity over the past week (43). The MADRS *Suicidal Thoughts* item served as the primary measure of suicidal ideation and was rated on a 7-point scale from 0 (“enjoys life”) to 6 (“explicit plans for suicide”). No participants endorsed the highest severity level in this sample (38). This item provides a state-like measure of suicidal ideation that complements the trait-like information captured by lifetime SA history (44).

### Study Design Overview

The study had four primary aims: 1) to construct PANSS symptom networks and identify symptom communities; 2) to link suicidality to PANSS symptom structure and identify candidate intervention targets; 3) to conduct simulation-based, symptom-targeted interventions using a stratified design; and 4) to evaluate intervention effects on both network structure and suicidal outcomes. All analyses were conducted in R (version 4.3.3).

### Construction of PANSS Network and Community Identification

PANSS data were modeled as a 30-node partial correlation network estimated using graphical Lasso regularization with EBIC model selection to reduce spurious edges (45). Networks were estimated and visualized using the *qgraph* package (46). Network stability was evaluated using case-dropping bootstraps (*bootnet*). Correlation stability (CS) coefficients exceeded recommended threshold of 0.50 (25, 47) (structure = 0.67; centrality = 0.70), indicating robust network estimation.

Symptom communities were identified using Exploratory Graph Analysis (EGA; *EGAnet*) (48, 49). Cluster solutions were validated using multidimensional scaling and PERMANOVA (Omnibus F = 2.17, permutation p < 0.0001) (50). We then created cluster-level PANSS networks by combining symptoms within each identified community into a single summary node. To examine links with suicidality, we added the Suicidal Thoughts item as an additional node to form the suicide-PANSS network. Bootstrap procedures were used to estimate 95% confidence intervals for network *edge weights* (i.e., connection strengths between nodes). When statistically evaluating multiple edges within the same network, p-values were adjusted using false discovery rate (FDR) correction (51).

#### Identification of Candidate Intervention Targets

Candidate symptom targets were identified using convergent network evidence (35, 36, 52). We first evaluated item- and cluster-level networks to identify symptoms with high influential potential within the PANSS network, focusing on four centrality metrics (33, 34, 50): 1) node strength (overall connectivity), 2) expected influence (incorporating the directionality of edges based on partial correlations), 3) closeness (how quickly a symptom can affect others), and 4) betweenness (quantifying how often a node lies on the shortest paths between others) (18). Centrality values were standardized as proportional deviations from the network mean to facilitate comparison. In addition, the magnitude and direction of edges linking Suicidal Thoughts to PANSS items and clusters were examined. Together, these metrics informed the selection of symptoms with strong potential influence on suicidality pathways.

#### Simulation of Symptom-targeted Intervention

We implemented a digital simulation framework to emulate randomized controlled trials (RCT) using empirically estimated symptom networks. Analyses were restricted to the 313 participants with complete PANSS, SA history, and MADRS Suicidal Thought data (**Supplemental Fig. 1**). This sample size is consistent with prior schizophrenia network studies demonstrating stable estimation under regularized network models (53, 54). Baseline PANSS profiles defined the pre-intervention state (T0).

### Stratification by Suicide Attempt History

Because lifetime suicide attempt history reflects a stable trait-like vulnerability to suicidality and is commonly incorporated in predictive models of suicide risk (5, 42, 55), we examined whether PANSS symptom severity and network structure differ between individuals with a history of suicide attempt (SA+; *N* = 107) and those without (SA−; *N* = 206) (56–58). As is common in clinical samples, the SA+ subgroup was smaller; therefore, subgroup networks were estimated using the fused graphical LASSO (*EstimateGroupNetwork*), which improves stability through joint estimation and partial pooling across groups (58).

Networks were compared at three levels: 1) PANSS item-level networks; 2) PANSS cluster-level networks, and 3) suicide-PANSS cluster network. In addition to global comparisons using the Network Comparison Test (*NetworkComparisonTest* package) (59), local network topology was evaluated descriptively using centrality metrics. Within-cluster connectivity was summarized as the average node strength within each cluster to characterize differences in the relative importance of symptom domains between SA + and SA− individuals. Cross-domain organization was further examined using bridge edge weights and cross-domain Spearman correlations.

### Emulated Randomized Controlled Trial Design

An emulated randomized controlled trial was conducted for the full sample, with suicide attempt (SA) history incorporated as a stratification factor to reduce confounding (59). Within each stratum, participants were randomly assigned (1:1) to a simulated intervention arm or a synthetic control arm. This design allowed estimation of simulated intervention effects and evaluation of whether responsiveness differed by SA history (22).

#### Simulated Deactivation (Intervention) Arm

Participants assigned to the intervention arm underwent targeted perturbation of selected symptom clusters. Symptom severities were reduced in proportion to their normalized expected-influence centrality scores, such that symptoms with greater network influence received larger reductions while the underlying network topology was preserved. The resulting modified PANSS profiles were treated as post-intervention outcomes (T1). Full algorithmic details are provided in the **Supplement**.

#### Synthetic Control Arm

Control-arm PANSS profiles at T1 were generated using a copula-based resampling approach that preserved the joint distribution of PANSS items (60). Specifically, PANSS items for control subjects were transformed using empirical cumulative distribution functions, resampled from the fitted copula, and back-transformed to the original scale. Fidelity of the synthetic control data was verified by comparing observed and simulated distributions (**Supplemental Fig. 2**).

### Evaluation of Intervention Effects

A multi-class neural network classifier (*neuralnet* package) was trained on the full baseline sample to predict MADRS Suicidal Thoughts from PANSS items, using a 70/30 train-test split with 10-fold cross-validation (61). Neural networks were selected to capture nonlinear relationships between the 30 symptom scores and suicidal ideation. The trained model was then applied to post-intervention PANSS profiles (T1) from both the intervention and control arms to generate predicted suicidal ideation levels.

Pre- to post-intervention changes in network structure were examined to confirm successful emulation of the intervention and control conditions. Cluster-level symptom networks were prioritized to ensure favorable sample-size-to-node ratios, and uncertainty in edge estimates was quantified using 95% bootstrap confidence intervals. Intervention effects were evaluated within each suicide-attempt (SA) stratum by comparing predicted suicidal ideation outcomes between the intervention and control arms, with group difference assessed using Wilcoxon-Mann-Whitney test, which is more robust to ordinal or non-normal outcomes (62). Sensitivity analyses were conducted to evaluate the robustness of the primary findings.

## Results

### PANSS Symptom Network Revealed Five Clusters

**Figure 1:**
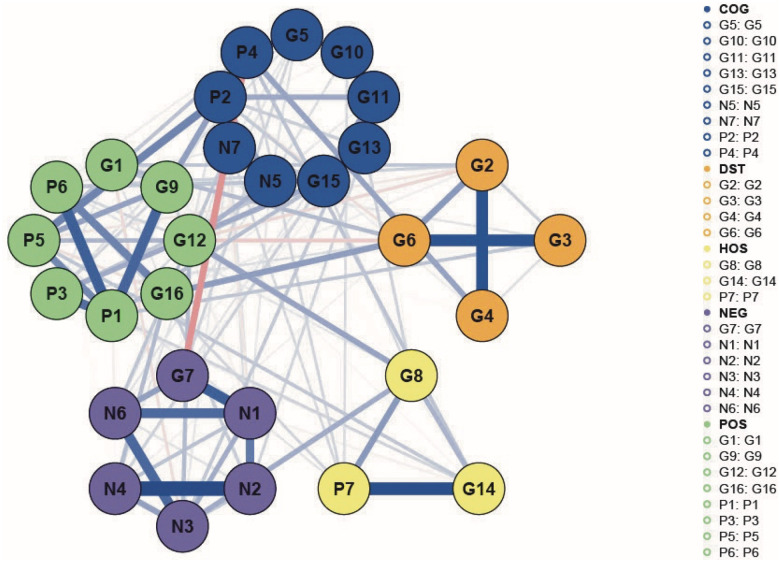
The PANSS symptom network of 30 items. Nodes represent individual PANSS items, and edges represent regularized partial correlations between symptoms. Blue edges indicate positive associations and red edges indicate negative associations; thicker edges correspond to stronger associations. Symptoms are grouped into five domains: Impaired Cognition-Control (COG), Distress (DST), Impulsive-Hostile (HOS), Positive/Impaired Salience (POS), and Negative/Withdrawn-Disengaged (NEG).

Exploratory Graph Analysis (EGA) applied to the full 30-item PANSS network (Fig. 1) revealed five coherent symptom clusters, consistent with prior factor-analytic models of schizophrenia spectrum disorders (39–41). Based on item content and theoretical structure, clusters were labeled as follows and referred to as *symptom domains* in what follows:

1. **Positive Symptoms (POS)**: P1 (Delusions), P3 (Hallucinations), P5 (Grandiosity), P6 (Suspiciousness/Persecution), G1 (Somatic Concern), G9 (Unusual Thought Content), G12 (Lack of Judgment and Insight), and G16 (Active Social Avoidance);
2. **Impaired Cognition-Control (COG)**: P2 (Conceptual Disorganization), P4 (Excitement), G5 (Mannerisms and Posturing), G10 (Disorientation), G11 (Poor Attention), G13 (Disturbance of Volition), G15 (Preoccupation), N5 (Difficulty in Abstract Thinking), and N7 (Stereotyped Thinking);
3. **Impulsive-Hostile (HOS)**: P7 (Hostility), G8 (Uncooperativeness), and G14 (Poor Impulse Control);
4. **Distress (DST)**: G2 (Anxiety), G3 (Guilt), G4 (Tension), and G6 (Depression);
5. **Negative Symptoms (NEG)**: G7 (Motor Retardation), N1 (Blunted Affect), N2 (Emotional Withdrawal), N3 (Poor Rapport), N4 (Passive/Apathetic Social Withdrawal), and N6 (Lack of Spontaneity and Flow of Conversation).

The network-derived clusters aligned closely with the established five-factor PANSS structure (33). Minor deviations, such as the grouping of G1, G12, and G16 with the Positive domain (rather than with Mood, Cognitive, or Negative, **Supplementary Table 1**), likely reflect empirical co-activation patterns that are captured more precisely in network-based approaches (48).

### Distress as the Primary Bridge Linking Psychopathology to Suicidality

Distress symptoms showed the most consistent and direct associations with suicidality across multiple analytic levels. In the PANSS-suicide network (**Supplemental Fig. 3a**), Suicidal Thoughts displayed positive item-level edges with G6 (Depression) and G3 (Guilt), both within the Distress domain. Critically, the strongest and only edge that remained significant after false discovery rate (FDR) correction was between Suicidal Thoughts and G6 Depression (edge = 0.147; 95% CI: [0.066, 0.264]). At the cluster level, Suicidal Thoughts was significantly associated exclusively with the Distress domain (edge = 0.292; 95% CI: [0.180, 0.401]), whereas no other symptom domains demonstrated FDR-significant links. Consistent with these network-based findings, higher levels of suicidal ideation (levels 4–5) were associated with greater Distress severity (**Supplemental Fig. 3b**).

Distress-related symptoms also demonstrated high centrality at the item level within the PANSS network. Although the Distress cluster did not rank highest in cluster-level centrality, several constituent symptoms emerged as influential or bridge nodes. Specifically, G2 (Anxiety) and G6 (Depression) demonstrated elevated strength (+ 6.9% and + 22.0% above the network average), while G4 (Tension) and G6 showed higher betweenness (+ 8.3% and + 24.4%), indicating greater involvement in network connectivity and information flow.

Taken together, these convergent findings identify Distress as a statistically supported and mechanistically plausible pathway linking psychopathology to suicidality, motivating its selection as the primary target for subsequent simulation-based intervention analyses.

### PANSS Network Differs by Suicide Attempt History Prior to Simulation

As shown in Table 1, participants with (SA+) and without (SA–) suicide attempts differed demographically: the SA+ group was older (median = 32 vs. 23 years) and included a higher proportion of females (39% vs. 25%).

Despite comparable overall symptom severity between groups (median PANSS: 68 in SA + vs. 67.5 in SA−), the Distress severity was significantly higher in individuals with prior suicide attempts (p < 0.01). In contrast, SA+ participants showed lower severity in Negative symptoms (p = 0.01) and were less impaired in Cognition-Control symptoms (p = 0.02, Table 1).

### Local Network Differences

**Figure 2:**
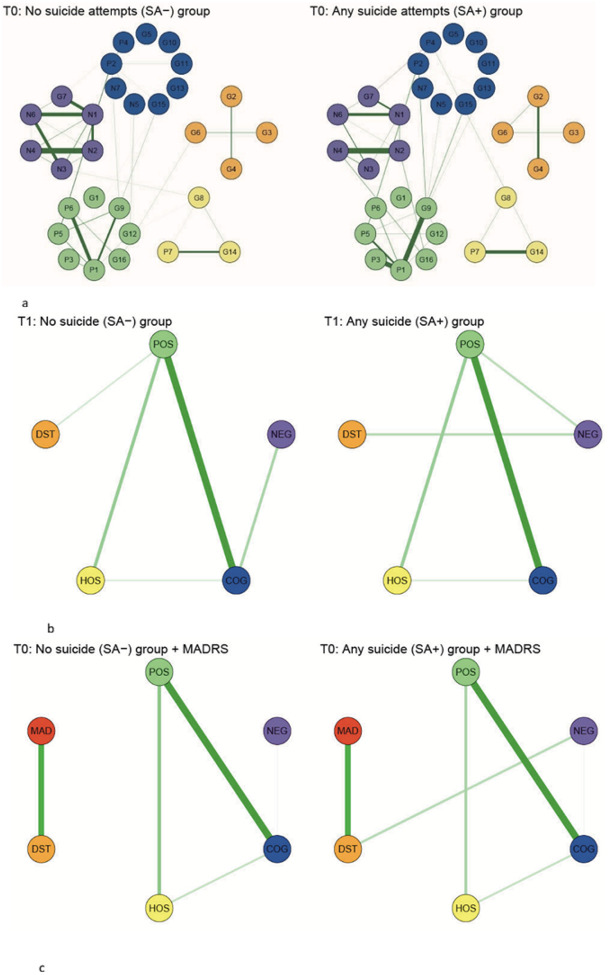
Pre-intervention (T0) network differences by suicide attempt history. Symptom networks are shown separately for individuals without a history of suicide attempt (SA−) and those with a history of suicide attempt (SA+). a. Item-level PANSS network. b. Cluster-level network aggregated across the five PANSS domains. c. Cluster-level network with the MADRS Suicidal Thoughts item added. All networks are estimated using baseline (T0) PANSS data. Edges represent partial correlations.

Beyond symptom severity, global network strength and global structure did not differ significantly between SA + and SA− groups (p = 0.222 and p = 0.344, *NetworkComparisonTest*). However, local network topology showed some divergence. Descriptively, the SA+ subgroup exhibited stronger within-cluster connectivity in the Positive, Impulsive-Hostile, and Distress domains (increases up to + 33%), alongside weaker connectivity within the Negative and Cognition-Control domains (approximately − 23%), consistent with the lower symptom severity observed in these latter domains (Fig. 2a).

### Cross-Domain Bridging Patterns

A further descriptive difference between groups was the presence of a cross-domain bridging edge between the Negative and Distress observed only in the SA+ group (edge = 0.237; Fig. 2b). Although this edge did not reach statistical significance, it corresponded to a substantially stronger cross-domain correlation in SA+ (Spearman’s Rho of Negative-Distress = 0.303, p = 0.001) than in SA− (Rho = 0.030, p = 0.663).

When Suicidal Thought was incorporated into the network, the Negative-Distress connection remained descriptively evident in the SA+ subgroup (edge = 0.121; Fig. 2c) but was absent in SA−. This pattern was further supported by partial correlation analyses controlling for Suicidal Thoughts, which indicated a moderate association between Negative and Distress domains in SA+ (partial Rho = 0.278, p < 0.05) but not in SA− (partial Rho = 0.012, p = 0.884).

Taken together, these findings suggest that individuals with and without a history of suicide attempts differ not only in symptom severity profiles but also in exploratory network organization. Accordingly, simulated interventions were evaluated separately within each stratum to mitigate potential bias arising from structural heterogeneity between groups (63).

### Clinical Prediction Demonstrated the Translational Potential of Digital Trials

To evaluate clinical relevance, we used supervised learning to predict suicidal ideation under simulated intervention and control conditions.

A neural network classifier was trained on the full sample (N = 313) using baseline PANSS item scores (T0) and MADRS Suicidal Thought. The model achieved a cross-validated test accuracy of 0.85 with 10-fold cross-validation (**Supplemental Fig. 4**). By capturing nonlinear relationships between multidimensional symptom profiles and suicidal ideation, the trained model was subsequently applied to post-intervention PANSS profiles (T1) to generate predicted suicidal ideation outcomes under each simulated condition.

### Network-level Change in the Full Sample

**Figure 3:**
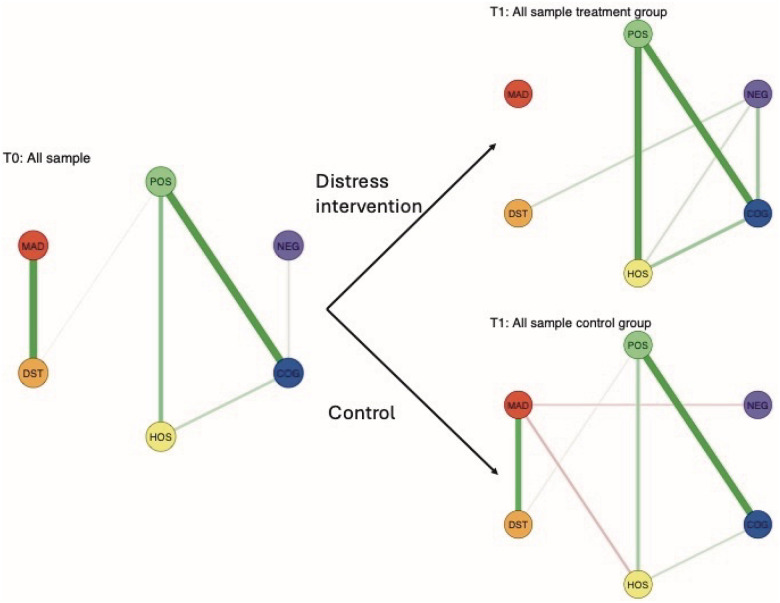
Cluster-level symptom networks pre- (T0) and post-intervention (T1) by treatment arm. Cluster-level networks including the MADRS Suicidal Thoughts item are shown at baseline (T0) and separately for the intervention and control arms post-intervention (T1). Changes in network structure illustrate differential symptom-suicidality associations following simulated Distress-targeted modulation.

At the network level, distinct post-intervention patterns emerged between the intervention and control arms. In the intervention arm, the association between Suicide and Distress was no longer present at T1 (edge = 0, 95% CI: [− 0.612, 0.445]), whereas it remained positive in the control arm (edge = 0.362, 95% CI: [0, 0.437], see Fig. 3).

In contrast, the control arm exhibited the emergence of weak negative associations between Suicide and Negative symptoms (edge = − 0.098, 95% CI: [− 0.669, 0]) and between Suicide and Hostility (edge = − 0.142; 95% CI: [− 0.502, 0]), patterns not observed in the intervention arm. A stratified network comparison is provided in **Supplemental Fig. 5**.

### Predicted Clinical Effects Stratified by Suicide Attempt History

#### SA+: Distress Modulation Reduced Predicted Suicidality

**Figure 4:**
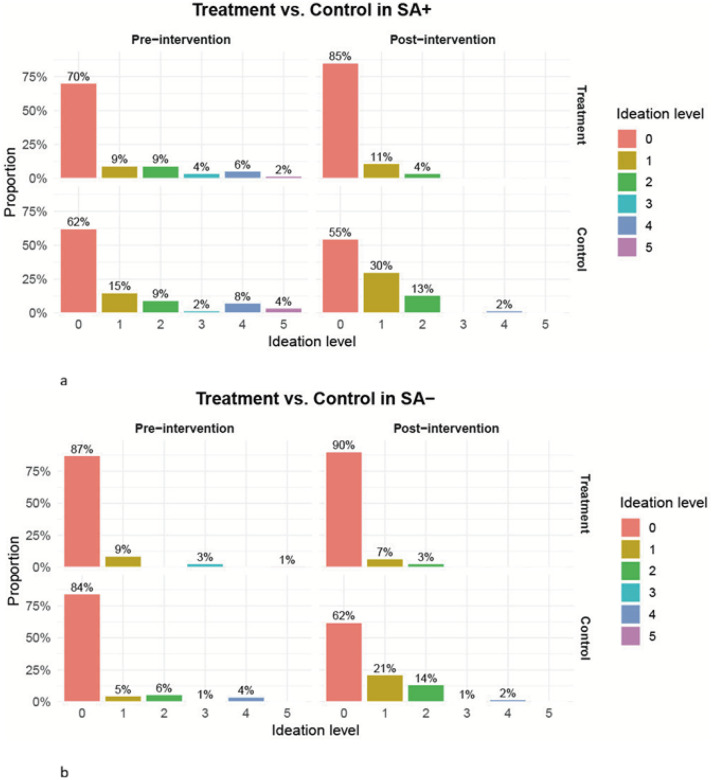
Distribution of predicted suicidal thoughts pre- and post-intervention, stratified by suicide attempt history. Histograms show the predicted distribution of suicidal thoughts at baseline (T0) and post-intervention (T1) for a. individuals with a history of suicide attempt (SA+) b. individuals without a history of suicide attempt (SA−). The outcome is the 6-level (0–5) *Suicidal Thoughts* item from the Montgomery-Åsberg Depression Rating Scale (MADRS), where higher scores indicate greater suicidal ideation. Post-intervention outcomes were generated using a two-layer neural network trained on baseline data, and proportions at each level are displayed.

Among individuals with a history of suicide attempt (SA+), simulated Distress-targeted modulation was associated with favorable shifts in predicted suicidal ideation. Under the intervention condition, the proportion of individuals predicted to have no suicidal ideation (level 0, “enjoys life”) increased by 15%, whereas it decreased by 7% in the control condition. Predicted mild ideation (level 1) remained stable under intervention but increased substantially in the control arm (15% to 30%) (Fig. 4a). Consistent with these distributional shifts, overall suicidal ideation severity was significantly lower under intervention than control (Wilcoxon-Mann-Whitney test p < 0.001).

Although derived from simulated outcomes, these results suggest that targeted Distress modulation may preferentially reduce suicidal ideation among individuals at elevated baseline risk.

#### SA−: Intervention Prevented Deterioration

In the lower-risk SA− subgroup, simulated Distress modulation primarily exerted protective effects by preserving baseline levels of suicidal ideation. Predicted rates of no ideation (level 0) increased modestly under intervention (87% to 90%) but decreased substantially in the control arm (84% to 62%) (Fig. 4b). Correspondingly, the prevalence of any suicidal ideation remained stable in the intervention condition but increased substantially under control. Overall, suicidal ideation severity differed significantly between intervention and control (Wilcoxon-Mann-Whitney test p < 0.001).

Together, these results suggest that even among individuals without prior suicide attempts, targeted Distress reduction may help maintain clinical stability and prevent worsening suicidal ideation.

#### Sensitivity Analysis for Age Heterogeneity

Although age was associated with lifetime suicide attempt (SA) history (Table 1), treatment assignment was randomized within each SA stratum, with the intent of balancing age and other baseline covariates between treatment and control arms separately within the SA + and SA− groups. As a result, confounding of the above treatment-control contrasts estimated within strata is expected to be minimized. Accordingly, primary analyses focused on stratum-specific treatment effects on predicted suicidal ideation.

Covariate balance for age and sex was assessed within each stratum and in the full sample (**Supplemental Table 2**). While sex was well balanced between treatment and control, a moderate residual imbalance in age was observed. To address this, we conducted age-stratified sensitivity analyses, in which predicted suicidal ideation outcomes were further stratified by median age within each SA stratum (**Supplemental Fig. 6**). These analyses were intended to assess robustness to residual age imbalance rather than to test age as an effect modifier; therefore, observed differences are interpreted descriptively.

Under the simulated intervention, the proportion predicted to have no suicidal ideation increased among both younger and older SA+ individuals (21.1% and 8.6%, respectively), increased among younger SA− individuals (12.2%), but decreased among older SA− individuals (− 3.2%). In contrast, predicted no-ideation rates declined across age groups in the control condition. Overall, these age-stratified sensitivity analyses indicate that the direction and relative magnitude of the simulated intervention effects were broadly consistent across age groups, with larger absolute improvements observed among younger participants.

Importantly, the qualitative pattern of benefit under the simulated Distress-targeted intervention and deterioration under control was preserved across age strata, supporting the robustness of the primary stratified findings to residual age imbalance.

## Discussion

The current study applied a data-driven network approach to identify symptom dimensions most closely associated with suicidality in schizophrenia spectrum disorders (SSD) and evaluated the potential impact of targeting these symptoms through simulations. There were several important findings. First, PANSS-based symptom networks reproducibly organized into a five-domain structure, consistent with multidimensional models of psychosis (41). Second, distress-related symptoms showed the most consistent and direct associations with suicidality across analytic levels. Thus, our study identified distress symptoms as a clinically relevant domain for intervention prioritization. Third, simulation-based modulation of distress symptoms was associated with lower predicted suicidal ideation, suggesting that interventions focused on targeting distress may warrant further investigation as a translational strategy for suicide prevention in SSD (14, 64). Collectively, these findings illustrate the value of network-informed, simulation-based approaches for prioritizing symptom targets and advancing precision-oriented intervention strategies.

PANSS symptoms consistently coalesced into five coherent clusters, aligning with prior factor-analytic studies of psychosis (29, 39–41, 65). In contrast to latent-variable models, which posit unobserved constructs as the primary drivers of symptom covariance (66–68), the network perspective conceptualizes psychopathology as a system of interacting symptoms that may reinforce one another (17, 19, 28, 31, 54). The reproducibility of this five-domain structure supports the utility of network models for capturing symptom heterogeneity in SSD and provides a flexible framework for examining how specific symptom domains relate to clinically important outcomes such as suicidality (69–72).

When suicidality was added to the PANSS network, distress-related symptoms demonstrated the strongest and most consistent links to suicidality. The distress symptoms include depression, anxiety, guilt, and tension, which emerged as the most influential bridge nodes to suicidality. Our findings align with a prior longitudinal network study in people with psychosis that identified depression as a key bridging symptom to suicidality over time (24). Distress symptoms are a clinically important domain of symptoms associated with numerous negative outcomes. For example, individuals with schizophrenia spectrum disorders and comorbid anxiety disorders are known to exhibit elevated risk for suicide attempts and higher lethality of attempts, along with other negative clinical outcomes, including increased risk of substance use, lower social functioning, and lower quality of life (5, 12, 13, 15). The present study extends this framework by providing network-level evidence that distress-related symptoms may act as key conduits linking psychopathology to suicidality.

Targeted reduction of distress-related symptoms was associated with attenuated network connectivity involving suicidality and with lower predicted suicidal ideation. Our stimulation results highlight the potential impact of distress-targeted interventions on suicidality: reducing distress symptoms significantly reduced suicidality among people with SSDs. Recent suicide-focused psychosis interventions further contextualize these findings (79, 80). The simulated intervention helped identify the next step in *what* intervention will directly target distress-related symptoms in people with schizophrenia spectrum disorders. Cognitive-behavioral therapies for psychosis (CBTp), including worry-focused and affect-focused interventions (78), are particularly relevant, given their demonstrated efficacy in reducing anxiety, depression, and related distress (75). In a recent randomized controlled trial conducted by our group (NCT04748679), an eight-week worry-focused CBTp intervention yielded greater improvements in distress-related symptoms than in positive symptoms (− 20.5% vs. −10.1%) (76). Although this trial does not constitute a direct validation of the present simulations, it provides converging evidence supporting the clinical relevance of targeting distress-related processes.

One aspect to consider is which individuals would benefit the most from a distress-targetted intervention. Given the heterogeneity in SSDs, some individuals may benefit more from the distress-targeted intervention. Our sensitivity analyses address some factors that may contribute to an individual benefiting more from the distress-targetted intervention, including high distress symptoms, prior suicide attempts, and age. First, there may be a subtype of people with SSD with elevated distress symptoms. Our group recently identified a *distress subtype* of SSD characterized by heightened negative affect, neuroticism, schizotypal personality traits, and a poorer quality of life (77). This subtype aligns with the affective pathway to psychosis that proposes an individual has heightened stress sensitivity and anxiety prior to the onset of psychosis, which leads to a presentation of psychosis with high distress symptoms (78–80). Second, targeting distress symptoms should be prioritized in individuals with a history of prior suicide attempts. Prior suicide attempts represent a stable marker of elevated suicide risk in SSD (57, 58), and individuals with and without such a history may differ not only in overall risk level but also in the organization of psychopathology relevant to suicidality. Third, younger individuals with SSD may show more improvement. Our age-stratified sensitivity analyses suggested potential heterogeneity in the magnitude of simulated intervention effects. Larger absolute improvements in predicted suicidal ideation were observed among younger participants, particularly among those with a history of suicide attempts. Together, our results underscore the importance of considering heterogeneity in SSDs and identify potential factors that may influence treatment effects.

Several limitations should be acknowledged. First, although the sample size was smaller than that of some large first-episode psychosis network studies (24), the homogeneous recruitment and standardized clinical assessments enhanced interpretability, and network stability metrics indicated robust estimation (e.g., correlation stability, or CS coefficients > 0.5). Second, PANSS and MADRS ratings both reflect a one-week symptom window and may not capture the longer-term fluctuations of distress or suicidality. A third and related point is that the study was cross-sectional; future longitudinal studies will be critical for determining the temporal stability and predictive validity of distress-related pathways in schizophrenia spectrum disorders. Fourth, simulated interventions do not substitute for real-world randomized controlled trials, which remain the gold standard for establishing clinical efficacy. Finally, analyses were conducted at the group level; future work should extend these methods to personalized, longitudinal networks to evaluate individual-specific symptom trajectories and intervention responsiveness (81).

In summary, the current study demonstrates that distress-related symptoms are a critical link to suicidality in individuals with SSD. By integrating network analysis with simulation-based intervention modeling, our study provides a data-driven framework for prioritizing symptom targets and generating hypotheses for future clinical trials. Together, our findings support further empirical evaluation of interventions targeting distress symptoms as a strategy for reducing suicide risk and improving outcomes for individuals with schizophrenia spectrum disorders. Our hope is that this study lays the groundwork for future interventions to improve the lives of people with SSD.

## Supplementary Files

This is a list of supplementary files associated with this preprint. Click to download.

- SupplementalTableFigure.pdf

## Figures and Tables

**Table 1:** Patient Demographics and clinical information

	N (non-missing)	SA+ (N = 109)	SA- (N = 208)	p-value
**Suicide attempts**	317	1.00 *2.00* 3.00	0.00 *0.00* 0.00	<0.01
**Age of attempt**	102	16.92 *20.00* 26.00		
**Diagnosis**	317			<0.01
Schizoaffective		0.40 44/109	0.12 26/208	
Schizophrenia		0.48 52/109	0.42 88/208	
Schizophreniform		0.12 13/109	0.45 94/208	
**Age**	317	24.0 *32.00* 46.00	20.00 *23.00* 30.00	<0.01
**Sex:** Female	317	0.39 43/109	0.25 53/208	0.01
**Race**	317			0.10
American Indian		0.02 2/109	0.01 2/208	
Asian		0.00 0/109	0.01 3/208	
Black		0.28 30/109	0.39 82/208	
White		0.67 73/109	0.57 118/208	
More than one race		0.04 4/109	0.01 3/208	
**Ethnicity:** Not Hispanic or Latino	297	1.00 100/100	0.98 193/197	0.15
**Education years**	289	12.00 *12.00* 15.00	12.00 *13.00* 15.00	0.10
**Age of hospitalization**	96	17.00 *21.00* 28.00	18.00 *23.50* 30.08	0.32
**Clozapine:** yes	317	0.08 9/109	0.04 9/208	0.15
**Lithium:** yes	317	0.04 4/109	0.02 5/208	0.52
**PANSS** total	317	53.00 *68.00* 76.33	56.00 *67.50* 81.00	0.37
POS	317	2.12 *2.88* 3.62	2.12 *2.69* 3.62	0.49
NEG	317	1.17 *1.67* 2.17	1.33 *2.00* 3.33	0.01
COG	317	1.44 *1.89* 2.33	1.56 *2.00* 2.67	0.02
HOS	317	1.00 *1.00* 1.67	1.00 *1.00* 1.86	0.10
DST	317	2.00 *2.50* 3.25	1.50 *2.00* 2.50	<0.01
**Outpatient: yes**	313	0.307 63/205	0.315 34/108	0.89

*N*: the number of participants with non-missing data. SA+: participants with a lifetime history of suicide attempt; SA−: participants with no history of suicide attempt. POS = Positive/Impaired Salience; COG = Impaired Cognition-Control; HOS = Impulsive-Hostile; DST = Distress; NEG = Negative/Withdrawn-Disengaged. Domain scores from POS to DST were computed as the average of item scores within each domain to account for unequal numbers of symptoms across domains. For continuous variables, the first quartile, median, and third quartile are reported; for categorical variables, percentages are reported. Group comparisons were performed using Pearson’s chi-squared tests for categorical variables and Wilcoxon rank-sum tests for continuous variables.
